# Internet-based cognitive behavioral therapy in children and adolescents with obsessive-compulsive disorder: A randomized controlled trial

**DOI:** 10.3389/fpsyt.2022.989550

**Published:** 2022-10-18

**Authors:** Karsten Hollmann, Carolin S. Hohnecker, Anna Haigis, Annika K. Alt, Jan Kühnhausen, Anja Pascher, Ursula Wörz, Rehan App, Heinrich Lautenbacher, Tobias J. Renner, Annette Conzelmann

**Affiliations:** ^1^Department of Child and Adolescent Psychiatry, Psychosomatics and Psychotherapy, University Hospital Tübingen, Tübingen, Germany; ^2^Section for Information Technology, University Hospital Tübingen, Tübingen, Germany; ^3^Department of Psychology (Clinical Psychology II), Private University of Applied Sciences, Göttingen, Germany

**Keywords:** obsessive-compulsive disorder (OCD), children, adolescents, internet-based psychotherapy, ambulatory assessment, videoconference, cognitive behavioral therapy (CBT), exposure with response prevention

## Abstract

**Objectives:**

Obsessive-compulsive disorder (OCD) in childhood and adolescence often leads to significant impairment in various areas of life and has a high risk of becoming chronic. Cognitive behavioral therapy (CBT) is the recommended first-line treatment, but it is too rarely implemented in accordance with guidelines and is often not available close to the patient’s home. Importantly, internet-based CBT could help to reduce this gap in care. Having previously successfully demonstrated the feasibility of an internet-based CBT approach, we aimed to assess its effectiveness in a waiting list controlled randomized trial.

**Methods:**

Children and adolescents aged 6–18 years with a principal diagnosis of OCD received 14 sessions of therapist-delivered CBT *via* videoconference distributed over 16 weeks. After inclusion, participants were randomly assigned to either the treatment or waiting list group. Participants in the treatment group began treatment immediately after baseline diagnostics, and participants in the waiting list group began treatment after a 16-week waiting period. The primary outcome was a pre-post comparison of OCD symptoms as measured with the Children’s Yale-Brown Obsessive Compulsive Scale (CY-BOCS). Additionally, remission was an important outcome measure. Follow-up assessments were conducted for all measures 16 and 32 weeks after completion of treatment.

**Results:**

A total of 60 children and adolescents were included into the analyses. Over the course of the treatment, OCD symptoms according to the CY-BOCS significantly decreased in the treatment group compared to the waiting-list control group. Cohen’s d between groups was 1.63. After the patients in the waiting list group also received the treatment, the OCD symptoms decreased significantly in this group as well. This improvement of symptoms increased over the course of the follow-up assessments. Remission rate peaked at the 32-week follow-up, with 68% in the treatment group and 79% in the waiting list group. Importantly, patient satisfaction with treatment was high to very high.

**Conclusion:**

In our study, OCD symptoms decreased significantly and remission rate was high after internet-based CBT. Those effects were comparable to those found in studies of face-to-face treatment. Although further evidence is needed, these are early indications that our approach may be a viable way to provide access to adequate treatment for children and adolescents affected by OCD.

**Clinical trial registration:**

[www.ClinicalTrials.gov], identifier [NCT05037344].

## Introduction

Obsessive-compulsive disorder (OCD) in childhood and adolescence is common with a prevalence of 1–3% (1–3). OCD characteristics are intrusive thoughts, urges, doubts, impulses and images that impose themselves on the individual against their will and cause strong unpleasant feelings, such as anxiety, discomfort, or distress. To reduce these feelings, patients with OCD frequently perform ritualized and repetitive actions that take up a large amount of time. Without adequate treatment, the course of OCD is usually chronic, and OCD symptoms may persist into adulthood (4, 5). OCD is associated with significant impairments in various areas of life (e.g., school, leisure time, friends, and family) (6), frequently resulting in a reduced level of psychosocial functioning (7). In addition, OCD has a high rate of comorbidity with other mental illnesses (8–10), contributing to the complexity of the disorder and its treatment.

Various studies (11, 12) have shown that starting treatment as soon as possible after the initial manifestation of OCD is important to avoid a chronic course. For this reason, it is vital that the disease is diagnosed early and that interventions are initiated according to the guidelines. The treatment of choice for OCD is cognitive behavioral therapy (CBT) and it should include exposure exercises with response prevention (E/RP) as a core element (13, 14). In terms of reducing OCD symptoms, meta-analyses demonstrate between-group effect sizes (ES) for CBT of 1.20 (15) and higher (ES = 1.45) (16). It is recommended that the exercises are conducted accompanied by therapists and in the places where the OCD symptoms occur most frequently (13). In cases of severe OCD and significant impairment, a combined treatment of CBT and medication with Selective Serotonin Reuptake Inhibitors (SSRI) should be considered (13). Treatment with medication alone should only be used when psychological treatment is declined by children or adolescents with OCD and their families, or they are unable to engage in treatment (14).

Despite the clear evidence base for CBT, many patients are not treated with it, and even when they are, E/RPs are too rarely used (17). The main reasons for this are structural (lack of availability of CBT, especially in rural areas) (18), on the part of the treatment providers themselves (lack of experience and associated uncertainty in the implementation of E/RPs) (19, 20), and practical (considerable time and organizational effort to implement E/RPs) (19, 20). The latter is especially true for the therapeutic accompaniment of E/RPs in the patient’s home environment.

Internet-based psychotherapy could help overcome at least some of these barriers in several ways. First, it would eliminate long travel times for patients, enabling some to attend regular treatment appointments with OCD experts in the first place. In addition, it is conceivable that an internet-based approach could lower the inhibition threshold for seeking help, especially for patients who, due to their OCD, cannot leave their home environment or can do so only with considerable difficulty. Therapists would have the opportunity to accompany their patients *via* video conference during exposure exercises in the respective trigger situations at home, significantly reducing the high organizational and time costs (e.g., travel time). Specifically, internet-based psychotherapy *via* videoconferencing could lead to a more frequent application of E/RP in the home context, which are accompanied therapeutically. This could further improve the treatment efficacy.

Recently, contact restrictions during the COVID-19 pandemic highlighted the importance of expanding access to psychotherapy beyond the current levels (21). Preliminary study findings indicated that the pandemic was also associated with an increase in symptom severity among children and adolescents with an preexisting OCD (22), whereas being in therapeutic treatment had a protective effect (23). However, the continuation of psychotherapeutic interventions throughout the pandemic was only possible with the assistance of videoconferencing. In Germany, where our trial was conducted, the technical and legal framework was created in 2019 to allow video-based therapy and further technology-based interventions to be used in standard care (24, 25). Consequently, this increase in the digitalization of psychotherapy expanded the range of available therapies, and the basis for this expansion is the growth in existing evidence for internet-based therapy approaches.

Various technology-based CBT approaches exist for pediatric OCD, which, on a preliminary level, can be divided into internet-based (e.g., videoconferencing, mail, chat, online programs) and non-internet-based (e.g., telephone) CBT. In addition, the CBT approaches differ concerning their scope, whether there is contact with a therapist, and whether the therapy is conducted synchronously in time between therapist and patient. Video-based approaches allow exposure exercises to be accompanied by the psychotherapist in the home environment in real time on the screen. Two studies with children and adolescents have been conducted in this regard (26, 27). From these, initial indications of effectiveness have emerged. However, these need to be confirmed and extended, as the total number of patients studied is still quite small (*n* = 53). In addition, some of the results refer to a subpopulation (4–8-year-olds) (26). Of the more representative sample in terms of age, the stability of effects was measured only in a part (*n* = 14) and this only over the relatively short period of 3 months.

We conducted a study to test the feasibility of a novel internet-based CBT approach, whereby therapist-administered psychotherapy sessions took place *via* videoconferences (28). As a basis for this kind of treatment, an existing therapy manual (29) was transformed into a version that could be used online. In addition, various technical elements and devices were combined to an extent that went beyond the previous use of technology in studies on childhood and adolescent OCD. Specifically, ambulatory assessment was essential; using a smartphone application, patients and parents provided daily feedback on OCD symptomatology, mood, the involvement of other family members in the performance of the rituals and avoidance behavior, and other stressors (e.g., daily hassles). Another element of the treatment was the use of an online data cloud system where the therapy materials were made available to patients and their parents. Overall, the feasibility study showed that our approach worked well and was accepted by both patients and their parents. In addition, there was a “high” to “very high” level of satisfaction with the treatment, and a reduction in OCD symptoms was achieved. From the therapists‘ perspective, the accompaniment of E/RPs in the home environment was highlighted as very positive. Finally, the therapist rated the ambulatory assessment as very helpful as it provided a good overview of the patient’s progress during the week and allowed him to address specific events in more detail during the sessions.

As the overall innovative concept was found to be feasible and was well accepted by the families, the effectiveness of the approach will be examined in the current study. The results to date of video-based approaches for pediatric OCD, although promising, are affected by the limitations described above. Evidence that such an approach is effective in typical children and adolescents with OCD remains, in our view, inconclusive. Further initial evidence is warranted. We therefore decided to use a randomized controlled trial with a waiting list control group design. The treatment consisted of 14 therapy sessions *via* videoconference, distributed over 16 weeks. Our primary hypothesis was that OCD symptoms, measured with the Children’s Yale-Brown Obsessive-Compulsive Scale (CY-BOCS), would decline more in the group that begins treatment immediately after enrollment in the study than during the same period in the waiting list control group. In addition, we hypothesized that, after the waiting list control group also received treatment, their OCD symptoms would decrease significantly. Furthermore, we expected the treatment success to be maintained beyond the end of therapy, as assessed using two follow-up measurements conducted in both groups at 16 and 32 weeks after treatment completion.

## Materials and methods

### Study design

The study was a single-blinded wait list randomized controlled trial designed to demonstrate the effectiveness of internet-based CBT for children and adolescents with OCD. The participants were randomly assigned to either the treatment or waiting list group. Participants in the treatment group began treatment immediately after baseline assessment, whereas participants in the waiting list group began treatment after waiting period. The duration of the waiting period was 16 weeks, which corresponded to the duration of treatment in the treatment group.

For the treatment group, the primary and secondary outcomes were measured before randomization (baseline, t0), at post-treatment (week 16, t1), at follow-up I (week 32, t2), and at follow-up II (week 48, t3). For the waiting list group, the outcomes were also measured before randomization (baseline, t0), at the end of the waiting period (week 16, t1), at post-treatment (week 32, t2), at follow-up I (week 48, t3), and at follow-up II (week 64, t4).

The study was carried out at the Department of Child and Adolescent Psychiatry, Psychosomatics and Psychotherapy, University Hospital of Psychiatry and Psychotherapy, in Tübingen, Germany. The study was approved by the Ethical Committee of the Medical Faculty of the University of Tübingen (639/2018BO1 dated 09/18/2018). The trial was registered at the US National Institutes of Health (ClinicalTrials.gov) #NCT05037344.

### Participants

The participants were recruited primarily through the OCD outpatient clinic in Tübingen, which is located in the south of Germany. Recruitment support came from colleagues of an OCD outpatient clinic in Cologne, located approximately 400 km away from Tübingen in western Germany. The colleagues there made potential participants aware of the study and recommended contacting the clinic in Tübingen. The core element of participant recruitment was a campaign conducted in collaboration with the Department of Communication of the University Hospital Tübingen using Google AdWords. When the relevant search terms were entered, information about the study was shown, and families could access the landing page *via* a link. Information about the study was also provided on the homepage of the German Society for OCD. Furthermore, brochures about the study were sent to schools nearby, as well as to child and adolescent psychiatrists and psychotherapists in Tübingen.

Eligible participants were children and adolescents between the ages of 6–18 years with a primary diagnosis of OCD according to the Diagnostic and Statistical Manual of Mental Disorders, Fifth Edition (DSM-5), a Children’s Yale-Brown Obsessive Compulsive Scale (CY-BOCS) total score of ≥ 16, daily access to a broadband internet connection, a at least one legal guardian, especially a parent who was able to participate in the study, and the ability to read and write in German. Participants with psychiatric comorbidities were included if the comorbid disorder did not have a higher treatment priority than OCD; participants taking a psychotropic medication could also be included if the medication had been at a stable dose for the 6 weeks prior to the baseline assessment. When children and adolescents were enrolled in the study, parents were specifically told that drug treatment status must remain unchanged.

Participants were excluded if they had an IQ below 70, a psychiatric comorbidity that required initial treatment, such as anorexia nervosa with massive underweight, suicidal ideation, or such a degree of severity of OCD symptoms that the indication for full inpatient treatment existed. This was the case, for example, when school attendance was no longer possible. In addition, participants were excluded if they had a substance use disorder or if their family was psychologically distressed to the point that participation in the sessions and care of their children during the study was not possible. No other psychological treatment was allowed during participation in the study.

Autism spectrum disorder was not considered an exclusion criterion for the study as long as the OCD symptoms were clearly in the focus at the time of the screening/baseline assessment and the affected subjects were able to express a clear desire for change in relation to these symptoms.

After the families had contacted the study team by mail or telephone, an appointment was made with them for a telephone screening. The aim was to clarify to the extent possible whether the inclusion criteria were fulfilled and whether there were indications of the presence of exclusion criteria. In addition, the families received information about the treatment in the study and study design. Both the children and adolescents and at least one legal guardian/parent participated in this screening, which was conducted by a licensed psychotherapist. In the event of potential eligibility, the children and adolescents and at least one guardian were invited to the clinic in Tübingen for a detailed assessment. This initially consisted of an interview with a licensed psychotherapist, in which the focus was on an in-depth exploration of OCD symptoms, their impact on family life, and the final clarification of the children’s and adolescents’ motivation for therapy. In addition, the families were given the opportunity to clarify any unanswered questions they had regarding study design and CBT for OCD. All children, adolescents and guardians provided written informed consent to participate during this appointment. If no clear indications of fulfilled exclusion criteria emerged in the interview, another licensed psychotherapist, whose role in the study was solely to conduct the diagnostic assessment, took over and conducted baseline measurements with the children, adolescents and legal guardians/parents (see sections Primary and Secondary outcome measures - Clinician-rated). To avoid overtaxing the children and adolescents, this frequently occurred on a separate appointment. In addition, children and adolescents as well as parents completed various clinical questionnaires (see section secondary outcome measures—child- and parent-rated). If the inclusion criteria were fully met, random assignment to one of the two conditions was made. Subsequently, participants and their parents were informed of inclusion and group membership by the psychotherapist who had conducted the interview. Before the first therapy session, another appointment was held during which participants and parents received an introduction to the use of the technical equipment (a.o. tablet with videoconference program, smartphone with ambulatory assessment application, data cloud) by a research assistant.

Due to the COVID-19 pandemic, the entire assessment was conducted *via* videoconferencing beginning in the spring of 2020. Informed consent forms and questionnaires were exchanged by mail between families and the study team.

The participants were able to discontinue treatment at any time if side effects occurred or at their request and were subsequently assisted in identifying another treatment option.

### Randomization and masking

The randomization list was developed by our Institute of Clinical Epidemiology and Applied Biometry (IKEaB) and originally consisted of eight blocks of six participants each. The allocation ratio between the treatment and waiting list groups was 1:1, and stratification did not occur. Several participants dropped out, almost exclusively during the waiting period from the control group. To prevent the sample size of the waiting list group from falling below 20, two additional blocks of six subjects each were created, increasing the number of included participants to 60. The allocation ratio remained unchanged at 1:1. The randomization list was kept in an opaque envelope in a locked cabinet. After inclusion in the study, the participants received a participant number according to the order of their detailed assessment, which was used to indicate which group they had been assigned to in the list. Feedback regarding which of the two groups the participants had been assigned to was given to them by one of the psychotherapists from the study team. The diagnosticians were blinded to group membership at all data collection points and did not have access to the randomization list. The families were repeatedly reminded by the study team that they were not allowed to provide any information regarding their group membership during the diagnostic procedure.

### Interventions

Participants in both groups received 14 sessions of CBT *via* videoconferencing. We decided to schedule 16 weeks for this, as we had learned in our feasibility study that it is not always possible to conduct one therapy session per week due to external circumstances such as flu-like infection of the participant. Each therapy session was scheduled to last up to 90 min. Once again, the treatment guide we developed specifically for internet-based CBT was used, which is based on the CBT manual by Wewetzer and Wewetzer, (29) and used successfully in a previous pilot study. The core elements of this guide are therapeutically supervised exposures with response management, cognitive interventions, and family-centered interventions.

Similar to traditional CBT treatment for OCD, our internet-based CBT consisted of four phases. Phase I (session 1) included the establishment of a therapeutic relationship and psychoeducation on the topic of compulsions. This also included the creation of an explanatory model. Phase II (sessions 2–4) taught participants how exposures with response prevention work and prepared them for this. Other key content included creating distance from the content of the obsessions, initial cognitive interventions, and first steps to reduce the extent of involvement of other family members in the compulsions. The central elements of Phase III (sessions 5–12) were the implementation of E/RPs (part of each session from session 5 onward), in addition to cognitive interventions and family-centered interventions. The exposure exercises were supervised therapeutically on screen and subsequently performed independently by the participants several times between sessions. Finally, Phase IV (sessions 13–14) focused on relapse prevention.

The treatment providers were licensed psychotherapists with several years of professional experience and expertise in OCD in childhood and adolescence. They received supervision from the therapeutic head of study during weekly team meetings. The head of study was a licensed psychotherapist with a high level of expertise and practical experience in the treatment of children and adolescents with OCD due to several years of leading the special outpatient clinic for pediatric OCD. In addition, he is co-author of the German-language guideline for OCD.

All the therapy materials were stored in a password protected data cloud in separate folders for participants and parents. Another component of the treatment was the information that participants and parents submitted separately on a daily basis *via* the application. This information was related to OCD symptomatology and the resulting impairments in daily life, avoidance behaviors, mood, and daily stresses. In addition, *via* the application, the participants kept a log of the progress of their independently performed E/RPs, and the therapists had access to this data and could use it when preparing for the next session. For this purpose, the families received a smartphone secured by software so that only access to study-specific applications was possible. Furthermore, another application was used that connected the smartphone to a physiological wristband. Using this application, subjects were asked to set timestamps for various events (e.g., the start and end of E/RP, the time of going to bed and waking up).

## Technical equipment

All the families were provided with a smartphone and a tablet, both of which had been preconfigured by our department. The therapy sessions were conducted *via* videoconference using the program Vidyo^®^. We used the secured data cloud of our hospital for the storage of therapy materials. Physiological data were measured using the AS97 physiology wristband from Beurer, which recorded heart rate, activity level in the form of movement, and sleep quality data. The aim was to collect information regarding physical signs of stress, especially during E/RPs in children and adolescents with OCD. We aim to report on the analysis of these data in a separate article.

### Measurements

#### Primary outcome measure—Clinician-rated

The primary outcome measure was the CY-BOCS, considered the gold standard for the diagnostic assessment of OCD in children and adolescents (30). This is a semi-structured, clinician-administered interview that evaluates the severity of obsessions and compulsions across five dimensions (time occupied by symptoms, interference, distress, resistance and degree of control over symptoms). The total score is calculated using 10 items, with a maximum possible score of 40 points. The cut-off value for identifying clinically relevant obsessive-compulsive symptomatology is ≥ 16 points. Internal consistency was good for the Obsession and Compulsion Severity Scores (Cronbach’s alpha = 0.80 and 0.82), and the Total Score (Cronbach’s alpha = 0.90) (31). This also applies to the Test-retest stability for the Obsession and Compulsion Severity Scores (ICC = 0.70 and 0.76), and the Total Score (ICC = 0.79) (31). The intraclass correlations for the CY-BOCS Total, Obsession, and Compulsion Severity scores were 0.84, 0.91, and 0.66, respectively, suggesting good to excellent interrater agreement (32). *The Clinical Global Impressions* (CGI) (33) are ratings used by the clinician to rate the severity of psychopathology (CGI-Severity) on a scale of 1 (no symptoms) to 7 (extremely severe) and the change after treatment compared to the baseline (CGI-Improvement) on a scale ranging from 1 (very much improved) to 7 (very much worse). At *r* = 0.58, there is a substantial relationship between obsessive-compulsive symptom severity scores (measured *via* the CY-BOCS) and the global OCD syndrome severity (measured via the CGI–Severity scale) (34).

#### Secondary outcome measures—Clinician-rated

The *Schedule for Affective Disorders and Schizophrenia for School-Age Children Present and Lifetime Version* (K-SADS-PL), is a semi-structured, clinician administered interview that assesses a range of psychopathology in children and adolescents. Interrater agreement in scoring screens and diagnoses was high (range: 93–100%) (35). The *Children’s Global Assessment Scale* (CGAS) allows a clinician to assess participants’ overall level of functional strain. The rating ranges from 0 to 100, with higher values indicating a better level of social function. The inter-rater reliability was 0.84, and the test-retest reliability at 0.85 (36). Finally, the *Basic Intelligence Test Scale 2-Revised* (CFT 20-R) is a speech-free measure of fluid intelligence. Psychometric results revealed a good retest-reliability (*r* = 0.80–0.82) and high internal consistency (Cronbach’s alpha = 0.95) (37).

At all measurement time points, all clinician-rated measures were performed by diagnosticians blinded to group membership. All diagnosticians were licensed psychotherapists, had performed all outcome measures prior to the start of the study, and were experienced in their use.

#### Secondary outcome measures—Child- and parent-rated

The *Child Obsessive–Compulsive Impact Scale*—*Revised* (COIS-R) is a self-report and parent-report questionnaire designed to assess the impact of OCD symptoms on the psychosocial functioning of children and adolescents in home, social, and academic environments. Reliability was excellent for the parent-report total score (ICC = 0.81). The youth-report form yielded similar test–retest reliability for the total score (ICC = 0.89) (38).

The *Screen for Child Anxiety Related Emotional Disorders* (SCARED) is a self- and parent-report questionnaire that assesses symptoms of panic disorder, generalized anxiety disorder, separation anxiety disorder, and social anxiety disorder, in addition it assesses symptoms related to school phobia. For the total score and each of the five factors in the child and parent versions, the authors report good internal consistency (Cronbach’s alpha = 0.74–0.93), good test-retest reliability (intraclass correlation coefficients = 0.70–0.90), and moderate parent-child agreement (*r* = 0.20–0.47) (39).

The *Depression Inventory for Children and Adolescents* (DIKJ) assess emotional distress. Considering the diagnostic criteria of the DSM-5, the degree of depressive impairment was assessed with the help of 26 items. The internal consistency was high (Cronbach’s alpha = 0.92) (40).

The *Child Behavior Checklist* (CBCL/4–18), which is a parent-report scale, measures a wide range of child behavioral and emotional problems, as well as the *Youth Self Report* (YSR/11–18), which is a self-report scale for children and adolescents. For both measures, the internal consistency for the total score was Cronbach’s alpha = 0.93. The correlation between parent and child total scores was *r* = 0.33 in a clinical norm sample (41).

The *Questionnaire for the Measurement of Health-Related Quality of Life in Children and Adolescents* (KINDL) is available in a child- and a parent-report version (42). The subscales are physical well-being, emotional well-being, self-esteem, family, friends, and everyday functioning. These can be summed to obtain a total score. Psychometric results revealed a high degree of reliability (Cronbach’s alpha = 0.70 for most of the subscales). The *Ulm Quality of Life Inventory for Parents* (ULQIE) measures quality of life of parents of chronically ill children. The instrument contains the dimensions physical and daily functioning, satisfaction with the situation in the family, emotional distress, self-development, and wellbeing. Cronbach’s alpha for the subscales = 0.75–0.88; for the global scale = 0.91. Retest reliability was between 0.69 and 0.86 (43).

#### Measurements of satisfaction, feasibility, and implementation

The *Client Satisfaction Questionnaire-8* (CSQ-8) was completed at post-treatment to assess the participant’s perceptions of the value of the services received (44). The questionnaire consists of eight items answered on a four-point Likert scale ranging from one to four. The total score ranges from 8 to 32, with higher scores indicating more satisfaction. The internal consistency was found to be 0.93 (45). We developed our own *Final Therapy Evaluation Questionnaire* based on relevant research as a measure of treatment evaluation (46, 47) for the child, the parents, and the therapist. Each item was rated on a four-point Likert scale, including the response options “I agree,” “I somewhat agree,” “I somewhat disagree,” and “I disagree.” This questionnaire covered questions regarding satisfaction with the therapy and aspects of implementation, such as adherence (intervention was delivered as intended—answered only by the therapist), quality (how well different program components were conducted), and program differentiation (unique features of the program).

The *Summary Therapist Feedback Form* (STFF) was conducted after treatment, with responses provided on a seven-point Likert scale ranging from “Not at all” to “Somewhat” to “Very much” in response options. This feedback form focused on therapists’ feedback regarding the user-friendliness of the therapy materials, the comprehensibility, the practicability of the treatment manual, and whether all essential treatment elements were included in the manual (44).

### Adverse events

In each therapy session, the therapists obtained an impression of the general emotional state of the participants and the extent of their OCD symptoms. If there were indications of a deterioration, contact was immediately made with the head of the study to discuss the further procedure and, if necessary, to initiate action (e.g., inpatient admission). If the situation was not acute, it was discussed at the weekly team meeting. If there was any uncertainty regarding the urgency, the head of study could be contacted at any time.

### Sample size

Existing studies were consulted for guidance on effect sizes, based on comparisons of CY-BOCS total scores. In a CBT for children and adolescents conducted *via* webcam (27), the effect size between treatment group (*n* = 16) and waiting list group (*n* = 15) for the treatment effect was *d* = 1.36. The effects in decreased CY-BOCS scores remained stable at 3-month follow up assessment. Another study on pediatric OCD (48), which compared a face-to-face exposure treatment (*n* = 10) with a waiting list group (*n* = 10) yielded an effect size of *d* = 1.23 between these two conditions at post-treatment assessment. The changes remained stable during the follow-up period, which averaged 14 weeks. In our feasibility study (28), in which we had used the same approach as in this RCT, the effect size pre-post-treatment was *d* = 2.02 at *N* = 9. We therefore knew that our approach was very likely to lead to symptom reduction.

Power calculations should take into account that the planned analysis of treatment effectiveness in this study will be a mixed ANOVA with group as the between-subject factor (treatment group; waiting list group) and time as the within-subject factor (t0 = baseline; t1 = end of treatment/end of waiting period). The interaction effect group × time was particularly important for the treatment evaluation and should therefore have had enough power. Another consideration was that the sample should be large enough to allow secondary analyses in follow-up analyses on individual courses and subgroup effects, and to obtain sufficient data in follow-up assessments. Regarding potential drop-outs, we were guided by another technology-based study, in which the follow-up duration was 12 months (49). Already at 6-month follow-up, up to 30% of the participants no longer participated in the assessments.

Assuming a large effect size (η^2^ = 0.15), an alpha-error of *p* = 0.05, and a 1-beta-error of 0.8, the total sample size was estimated by 48 (i.e., 24 per group). The goal was to have at least 20 participants per group at the end of treatment in both groups (t2). According to drop-outs during the waiting period (9 of eligible 24 participants) and to prevent the sample size of the waiting list group from falling below 20 and to gain enough data for the follow-up assessments, it was necessary in the course of the study to increase the number of included participants to 60 (*n* = 30 in each group).

### Data processing and statistical analysis

The data were analyzed using R (Version 4.0.0) and IBM SPSS Statistics (Version 27). All randomized participants were included in the analyses, in accordance with intention-to-treat principles (50). For various reasons, results were not available for all participants for all measurement time points. For example, families who dropped out of the study before the start of treatment, were mostly no longer willing to participate in diagnostic appointments.

Regarding the handling of missing values, the recommendations of the National Research Council (51) were followed. In a first step, an analysis of the missing data was performed and found that not all of the missing data fulfilled the Missing Completely at Random Criterion (MCAR) (52). Data were analyzed to determine if missing values correlated with any baseline characteristics (i.e., group, sex, age, comorbidity, duration of OCD symptoms) or missing values of other measures *via* chi-square tests und logistic regressions. Subsequently, considering the variables associated with the pattern of missing data, multiple imputations for interval scaled outcome measures were performed. Fifty new data sets were created for each outcome measure for each of the measurement time points t1 to t4. An exception is the assessment of whether participants met the criteria for remission and/or response. No imputation was performed for this categorical assignment, and only participants who had received the full treatment were included.

Both data sets (original and imputed data) were analyzed in the below mentioned manner. Importantly, regardless of which of the two data sets was used, there were no differences in the results regarding the effectiveness of the treatment and the stability of the treatment effects.

Presented are the analyses of the imputed data. An overview of the original primary and secondary outcome measures can be found in Appendix Table A1.

Differences between the two groups at t0 were calculated using *t*-tests and chi-square tests. If necessary, the degrees of freedom in the *t*-tests were Welch corrected. For all primary and secondary outcomes norm values were used, if available.

Analyses were done in two steps for interval scaled measures. In the first step, the effectiveness of the treatment was assessed. Mixed ANOVAs with group as the between-subjects factor (treatment group; waiting list group) and time as the within-subjects factor (t0 = baseline assessment; t1 = end of treatment/end of the waiting period) were calculated.

In a second step, the stability of the treatment effects found in the first step were examined. Mixed ANOVAs were calculated with group as the between-subjects factor (treatment group; waiting list group) and time as the within-subjects factor (post-treatment, follow-up I, follow-up II). To establish a calculation basis for the comparison of the two groups with regard to the follow-up values after completion of the treatment, the results for all primary and secondary outcome measures were combined in the SPSS matrix into three variables per outcome measure. For example, the CY-BOCS scores from measurement time point t1 for the treatment group and measurement time point t2 for the waiting list group formed the variable “CY-BOCS post-treatment,” the scores of t2 for the treatment group and those of t3 for the waitlist group formed “CY-BOCS Follow-Up I,” and the values at t3 for the treatment group and t4 for the waiting list group formed “CY-BOCS Follow-Up II.”

To further analyze significant results of the ANOVAS two-sided *t*-tests were conducted. For measures that were strongly hypothesis-driven (CY-BOCS, CGI-I) the alpha level was 0.05. For all others, it was set to 0.001, to reduce the risk of an alpha error.

Effect sizes (ES) were estimated for CY-BOCS total scores using Cohen’s *d.* These were calculated both between groups at measurement point t1 and within groups for the comparison of pre-treatment and post-treatment. If the standard deviations of the compared CY-BOCS means weren’t equal, pooled standard deviations where used.

Participants were classified as responders if they had at least a 35% reduction in total CY-BOCS scores compared with baseline measurements (t0) and if they had a CGI-Improvement value of 1 or 2. Remission was defined as a CY-BOCS total score of 12 or less and a CGI-Severity value of 1 or 2 after treatment was completed (53).

As all participants received the same treatment, there was no analysis by group in terms of the measurements of feasibility, acceptance, and implementation. For the CSQ-8, means and standard deviations were calculated across all participants, both for the individual items and the total score. For the STFF, means and standard deviations were calculated across all therapists for each item. The responses to the Final Therapy Evaluation Questionnaire were considered separately for participants and parents, and the frequency of agreement with the statements was determined as a percentage.

## Results

### Sample characteristics and study flow

**FIGURE 1:**
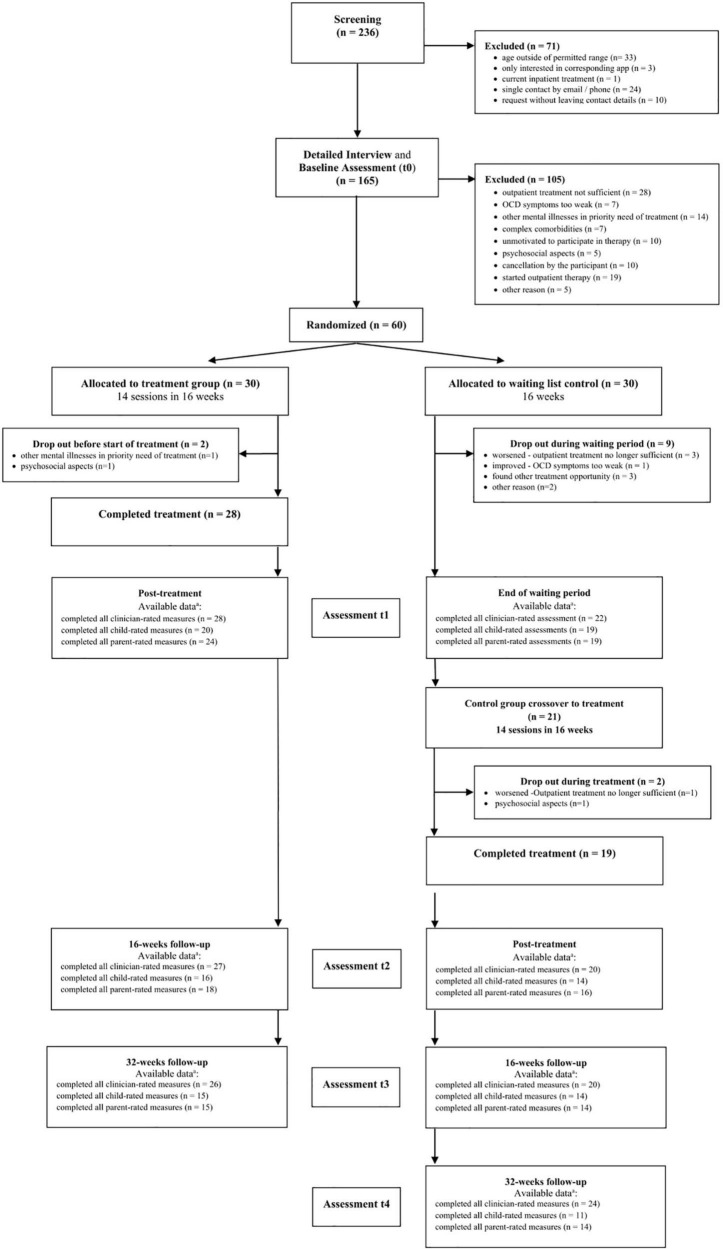
Study flow chart. Listed as available data ^a^ are all data available at that measurement point, regardless of whether participants received treatment or not.

Figure 1 displays the participant flow. A total of 236 families were screened for eligibility between January 2019 and November 2020.

60 children and adolescents in total were enrolled in the study and randomly assigned in equal numbers to one of the two conditions, making each group a total of 30 participants. In the treatment group, two participants dropped out of the study before beginning the intervention. The remaining 28 subjects began and all completed treatment. In the waiting list group, nine participants dropped out by the end of the waiting period, leaving the remaining 21 participants to begin treatment. During treatment, 2 subjects dropped out of the study, so it was still completed by 19 participants in the waiting group.

Data from all 60 participants at each measurement time point were included in the analysis of the original data, if available, even if they had not received or completed treatment. As shown in Figure 1, the number of participants who attended post-treatment follow-up visits varied.

Table 1 presents the baseline characteristics of the sample. The two groups did not differ significantly concerning any demographic or clinical variables, except for the mothers’ educational level. In the treatment group, the proportion of mothers with an academic degree was significantly higher than in the waiting list group. Of the participants, 60% were male (*n* = 36), and the mean age of all participants was 13.54 years (*SD* = 2.76). The average distance between the families’ homes and the study center in Tübingen was *M* = 171.9 km (*SD* = 151.5), and the median was Mdn = 132.0 km. The participants had experienced obsessive-compulsive symptomatology for an average of *M* = 31.03 months (*SD* = 30.76), and the median was Mdn = 20.00 months. At baseline assessment, 71.7% (*n* = 43) of the participants were diagnosed with at least one comorbid mental disorder. Overall, 31 participants (52%) had previously received psychological treatment, 22 had been treated with a psychotherapy other than CBT, and nine had been treated with CBT. E/RP had previously been used with two participants during their CBT treatment. In the other psychotherapy procedures, no E/RP against OCD symptoms had been conducted for any participant.

**TABLE 1:** Sociodemographic and clinical characteristics of participants at baseline (*N* = 60).

	Treatment group (*n* = 30)	Waiting list group (*n* = 30)	Statistical analysis regarding possible group differences
**Gender**			
Female/male	12 (40.0%)/18 (60.0%)	12 (40.0%)/18 (60.0%)	χ^2^ (1) = 0, *p* = 1.0
**Age (years)**			
Mean (*SD*) [range]	12.60 (2.88) [7–17]	13.87 (2.68) [7–18]	*t*(58) = 1.76, *p* = 0.084
**Migration background**			
Yes/no	5 (17.0%)/24 (80.0%)	5 (16.7%)/25 (83.3%)	χ^2^ (1) = 003, *p* = 0.953
**IQ**			
CFT 20-R mean *(SD)*	109.35 (13.52)	106.93 (11.62)	*t*(55) = 0.73, *p* = 0.470
**Parent educational level mother**			
Undergraduate degree or higher	22 (75.9%)	13 (46.4%)	χ^2^ (1) = 5.21, *p* = 0.022*^a^*
No academic degree	7 (24.1%)	15 (53.6%)	
**Parent educational level father**			
Undergraduate degree or higher	17 (63.0%)	14 (50.0%)	χ^2^ (1) = 0.939, *p* = 0.418*^a^*
No academic degree	10 (37.0%)	14 (50.0%)	
**Distance between patients’ residence and study site (km)**			
Mean *(SD)* [range]	173.46 (174.5) [10-771]	170.5 (127.5) [15–557]	*t*(58) = 0.08, *p* = 0.940
**Duration of OCD symptoms (months)**			
Mean *(SD)* [range]	28.20 (26.64) [3–105]	33.63 (34.21) [1–120]	*t*(58) = 0.69, *p* = 0.495
**Previous psychological treatment of OCD**			
Treatment: yes/no	16 (53.3%)/14 (46.7%)	15 (50.0%)/15 (50.0%)	χ^2^ (1) = 0.067, *p* = 0.796
CBT including E/RP	1 (3.3%)	1 (3.3%)	*^b^*
CBT without E/RP	6 (20.0%)	1 (3.3%)	*^b^*
Other	9 (30.0%)	13 (43.3%)	*^b^*
**Ongoing psychotropic medication**			
Medication: yes/no	2 (6.7%)/28 (93.3%)	3 (10.0%)/27 (90.0)	χ^2^ (1) = 0.183, *p* = 0.669
SSRI	2 (6.7%)	1 (3.3%)	*^b^*
Tricyclic antidepressants	0 (0.0%)	1 (3.3%)	*^b^*
Stimulants	0 (0.0%)	1 (3.3%)	*^b^*
**Number of participants with 0–3 comorbid diagnoses**			
Comorbid diagnosis: yes/no	20 (66.7%)/10 (33.3%)	23 (76.7%)/7 (23.3%)	χ^2^ (1) = 0.739, *p* = 0.390
One	10 (33.3%)	13 (43.3%)	*^b^*
Two	8 (26.7%)	6 (20.0%)	*^b^*
Three	2 (6.7%)	4 (13.3%)	*^b^*
**Frequency of comorbid diagnoses (K-SADS-PL)**			*^b^*
Depressive episode	2	5	
Anxiety disorders			
Specific phobia	8	10	
Social phobia	1	2	
Generalized anxiety disorder	4	8	
Separation anxiety	4	2	
Tic disorder	4	2	
ADHD	5	5	
Childhood emotional disorders with sibling rivalry	2	1	
Other childhood emotional disorders	1	1	
Depersonalization and derealization syndrome	1	0	
Autism spectrum disorder	0	1	

^a^The variables for calculation of the chi-square test were educational level (undergraduate degree or higher vs. no academic degree) and group (treatment group vs. waiting list group).

^b^No statistical analysis was performed due to the insufficient number of values per cell. *N* = 59 for migration background. *N* = 57 for IQ. *N* = 57 for mothers’ educational level. *N* = 55 for fathers’ educational level.

### Effectiveness of the treatment

#### Primary outcomes

##### Children’s Yale-Brown Obsessive Compulsive Scale^1^

**FIGURE 2:**
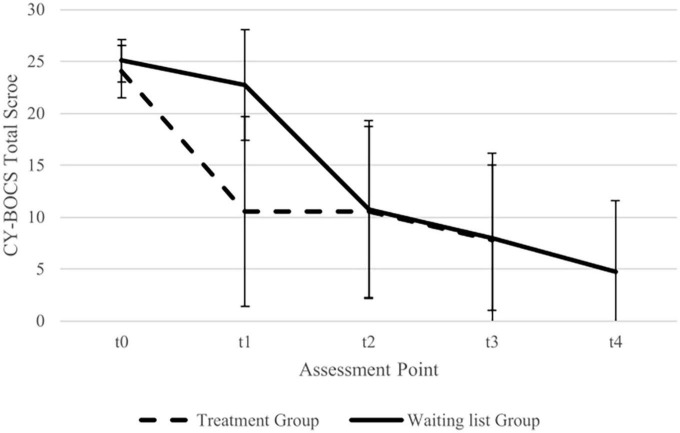
Imputed data for total Children’s Yale Brown Obsessive Compulsive Scale (CY-BOCS) mean scores (with standard deviation) for treatment group and waiting list group. Assessment points treatment group: t0 = baseline, t1 = post-treatment, t2 = 16 weeks Follow Up, t3 = 32 weeks follow-up. Assessment points waiting list group: t0 = baseline, t1 = pre treatment, t2 = post-treatment, t3 = 16 weeks follow-up, t4 = 32 weeks follow up.

A graphical representation of the CY-BOCS scores for every assessment point is shown in Figure 2 and a list of the individual scores for all outcome measures in Table 2.

**TABLE 2:** Imputed data for primary and secondary outcome measures.

Unadjusted mean ± standard deviation									
	Treatment group				Waiting list group				
Measure	Baseline assessment (t0)	Post-treatment (t1)	Follow-up I (t2)	Follow-up II (t3)	Baseline assessment (t0)	End of waiting period (t1)	Post-treatment (t2)	Follow-up I (t3)	Follow-up II (t4)
***Clinician-rated measures***									
CY-BOCS	24.03 ± 2.54	10.52 ± 9.15	10.51 ± 8.26	7.83 ± 8.35	25.07 ± 2.07	22.74 ± 5.32	10.76 ± 8.58	8.00 ± 7.01	4.75 ± 6.84
CGI-Severity	4.93 ± 0.52	2.35 ± 1.56	2.34 ± 1.52	2.05 ± 1.45	5.07 ± 0.37	4.59 ± 0.81	2.42 ± 1.51	1.99 ± 1.15	1.46 ± 0.85
CGI-Improv.		1.89 ± 1.17	1.81 ± 1.18	1.62 ± 1.02		3.86 ± 0.89	1.50 ± 1.15	1.35 ± 0.67	1.21 ± 0.42
CGAS	60.20 ± 10.48	82.10 ± 14.86	81.68 ± 11.68	83.96 ± 14.88	60.03 ± 10.05	65.40 ± 11.46	82.22 ± 13.31	85.87 ± 10.14	90.14 ± 10.08
***Child-rated measures***									
YSR	60.41 ± 20.08	48.18 ± 16.66	45.93 ± 17.73	42.69 ± 21.48	58.56 ± 18.50	53.75 ± 17.87	49.56 ± 18.40	46.71 ± 21.19	43.69 ± 21.50
SCARED	20.75 ± 13.25	14.02 ± 10.88	14.57 ± 10.11	12.48 ± 10.68	21.16 ± 11.55	17.45 ± 11.25	16.08 ± 10.67	14.49 ± 12.98	11.69 ± 11.07
COIS-RC	19.70 ± 17.04	8.01 ± 10.12	5.51 ± 8.92	4.88 ± 11.70	17.44 ± 10.86	12.17 ± 9.13	6.07 ± 8.11	3.84 ± 9.26	1.09 ± 8.51
DIKJ	14.20 ± 10.03	10.66 ± 8.58	8.36 ± 7.84	6.47 ± 7.67	14.81 ± 7.88	11.32 ± 8.38	9.20 ± 7.64	7.29 ± 6.91	6.22 ± 7.19
KINDL	70.40 ± 12.64	73.65 ± 11.40	72.72 ± 12.62	74.65 ± 12.69	68.94 ± 11.06	73.30 ± 11.56	75.90 ± 11.03	76.21 ± 12.25	78.38 ± 11.52
***Parent-rated measures***									
CBCL^a^	63.77 ± 7.75	56.80 ± 10.96	55.84 ± 10.08	52.05 ± 10.92	64.15 ± 7.27	60.84 ± 8.61	55.94 ± 8.90	52.82 ± 8.86	50.27 ± 8.59
SCARED	20.34 ± 13.11	15.85 ± 10.44	14.93 ± 10.67	12.43 ± 12.05	20.33 ± 8.53	17.46 ± 9.49	14.96 ± 10.60	11.67 ± 10.34	9.95 ± 9.21
COIS-RP	25.87 ± 18.10	12.10 ± 13.06	11.60 ± 17.69	7.66 ± 13.52	22.75 ± 12.14	15.92 ± 12.51	11.77 ± 16.38	6.42 ± 11.25	2.69 ± 11.13
KINDL	64.87 ± 12.65	72.08 ± 11.67	71.98 ± 12.12	75.24 ± 11.21	62.20 ± 14.00	68.54 ± 12.95	71.31 ± 10.18	76.80 ± 10.04	78.23 ± 11.10
ULQUIE	75.20 ± 16.78	75.17 ± 16.10	73.38 ± 21.31	77.33 ± 17.86	75.88 ± 15.26	81.58 ± 11.01	84.40 ± 13.44	86.18 ± 13.06	86.43 ± 15.77

^a^T-values.

A mixed two-way ANOVA with group (treatment group, waiting list group) as the between-subjects factor and time (t0, t1) as the within-subjects factor for CY-BOCS scores showed a significant effect of time [*F* (1, 58) = 68.47, *p* < 0.001], a significant effect of group [*F* (1, 58) = 39.22, *p* < 0.001], and a significant group x time interaction effect [*F* (1, 58) = 34.52, *p* < 0.001]. The significant interaction was further analyzed. In the treatment group, there was a significant difference between t0 and t1 [*t* (29) = 8.43, *p* < 0.001], whereas in the waiting list group, the difference was not significant [*t* (29) = 2.24, *p* = 0.067]. Between-group comparisons revealed a significant difference between the treatment group and the waiting list group for t1, with those in the treatment group showing lower CY-BOCS scores than those in the waiting list group [*t* (46.47) = 6.33, *p* < 0.001], whereas comparison between the groups was non-significant for t0 [*t* (55.70) = 1.73, *p* = 0.089].

Cohen’s *d* between groups for t1 was *d* = 1.63. The within-group effect size for the treatment group (t0 to t1) was *d* = 2.01.

##### Clinical global impressions-severity

A mixed two-way ANOVA with group (treatment group, waiting list group) as the between-subjects factor and time (t0, t1) as the within-subjects factor for CGI-Severity scores showed a significant effect of time [*F* (1, 58) = 76.87, *p* < 0.001], a significant effect of group [*F* (1, 58) = 51.39, *p* < 0.001], and a significant group x time interaction effect [*F* (1, 58) = 36.84, *p* < 0.001]. In the treatment group, there was a significant difference between t0 and t1 [*t* (29) = 8.41, *p* < 0.001]. This difference also existed in the waiting list group [*t* (29) = 2.92, *p* = 0.020]. Between-group comparisons revealed a significant difference between the CGI-Severity in the treatment group and the waiting list group at t1 [*t* (43.68) = 6.99, *p* < 0.001; treatment group < waiting list group], whereas comparison between the groups was non-significant for t0 [*t* (51.96) = 1.15, *p* = 0.256].

##### Clinical global impressions-improvement

After completed treatment (t1), the participant’s condition was rated as much better or very much better (CGI-Improvement value of “1” or “2”) in 22 of 28 participants (79%) in the treatment group. In the waiting list group this was not the case for any participant at the end of the waiting period.

##### Treatment remission and response

In the treatment group, 18 of the 28 participants (64%) who completed treatment met the remission criteria at the end of treatment (t1). In the waiting list group, none of the participants met the remission criteria after the end of the waiting period (t1), [X^2^ (1) = 19.80, *p* < 0.001]. At the same measurement time point, 22 of the 28 participants (79%) in the treatment group met response criteria. In the waiting list group this was not the case for any participant [X^2^ (1) = 28.07, *p* < 0.001].

#### Effectiveness of treatment in the waiting list group

After the participants in the waiting list group received treatment (t2), there was a significant decrease in CY-BOCS scores compared with time point end of waiting period (t1) [*t* (29) = 5.22, *p* < 0.001]. This change was also evident in the CGI-Severity scores [*t* (29) = 7.19, *p* < 0.001]. The participant’s condition was rated as much better or very much better (CGI-Improvement) in all participants, after receiving treatment (t2). The within-group effect size for the waiting list group (t1 to t2) was *d* = 1.64.

#### Secondary outcomes

An overview of the analysis results for all secondary outcome measures for the time points t0 and t1 can be found in Table 3. The significance level was set to *p* = 0.001 to account for multiple testing.

**TABLE 3:** Statistical analyses of secondary outcome measures—treatment effects.

	Mixed two-way ANOVA with the factors group (treatment, waiting list) and time (t0, t1)		
Measure	ME time	ME group	IA time × GROUP
*Clinician-rated measure*			
CGAS	***F*** (1, 58) = **47.87, *p*** < **0.001; t1** > **t0**	***F*** (1, 58) = **13.17, *p* = 0.001; TG** > **WG**	***F*** (1, 58) = **17.81, *p*** < **0.001** TG: *t* (29) = 7.02, *p* < 0.001; t1 > t0 WG: *t* (29) = 2.22, *p* = 0.053 t0: *t* (57.90) = 0.06, *p* = 0.950 t1: *t* (54.22) = 4.89, *p* < 0.001; TG > WG
*Child-rated measures*			
YSR	*F* (1, 58) = 12.14, *p* = 0.010	*F* (1, 58) = 0.41, *p* = 0.639	*F* (1, 58) = 2.84, *p* = 0.232
SCARED	*F* (1, 58) = 7.55, *p* = 0.019	*F* (1, 58) = 0.77, *p* = 0.436	*F* (1, 58) = 0.80, *p* = 0.466
COIS-RC	***F*** (1, 58) = **17.35, *p*** < **0.001; t1 < t0**	*F* (1, 58) = 0.25, *p* = 0.068	*F* (1, 58) = 2.63, *p* = 0.149
DIKJ	*F* (1, 58) = 11.36, *p* = 0.005	*F* (1, 58) = 0.19, *p* = 0.717	*F* (1, 58) = 0.34, *p* = 0.658
KINDL	*F* (1, 58) = 4.84, *p* = 0.047	*F* (1, 58) = 0.26, *p* = 0.670	*F* (1, 58) = 0.30, *p* = 0.674
*Parent-rated measures*			
CBCL^a^	***F*** (1, 58) = **15.76 *p*** < **0.001; t1 < t0**	*F* (1, 58) = 1.54, *p* = 0.260	*F* (1, 58) = 2.17, *p* = 0.201
SCARED	*F* (1, 58) = 6.27, *p* = 0.031	*F* (1, 58) = 0.21, *p* = 0.713	*F* (1, 58) = 0.54, *p* = 0.561
COIS-RP	***F*** (1, 58) = **25.34, *p*** < **0.001; t1 < t0**	*F* (1, 58) = 0.12, *p* = 0.799	*F* (1, 58) = 3.11, *p* = 0.125
KINDL	*F* (1, 58) = 13.18, *p* = 0.002	*F* (1, 58) = 1.39, *p* = 0.284	*F* (1, 58) = 0.23, *p* = 0.716
ULQUIE	*F* (1, 58) = 1.69, *p* = 0.277	*F* (1, 58) = 1.52, *p* = 0.293	*F* (1, 58) = 1.82, *p* = 0.279

^a^*T*-Values. Significant values, defined as *p* ≤ 0.001, are in bold. ME Time, Main Effect Time; ME Group, Main Effect Group; IA Time × Group = Interaction of Time × Group. TG, Treatment group; WG, Waiting list group.

Only the significant changes are described below, all other analyses revealed no significant effects, while descriptively results indicated a general improvement in mental health.

**FIGURE 3:**
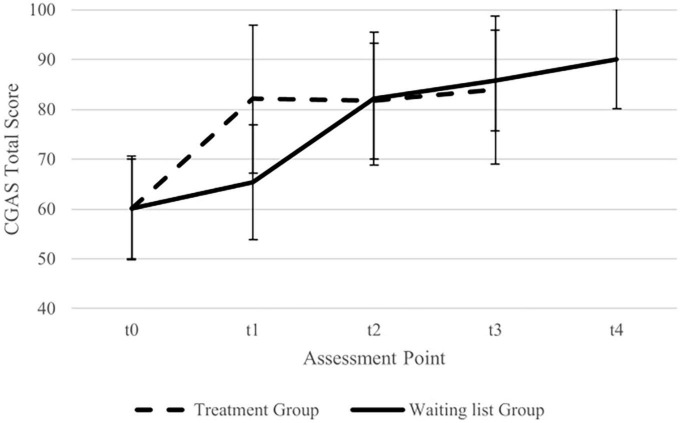
Imputed data for total Children’s Global Assessment Scale (CGAS) mean scores (with standard deviation) for treatment group and waiting list group. Assessment points treatment group: t0 = baseline, t1 = post-treatment, t2 = 16 weeks follow up, t3 = 32 weeks follow-up. Assessment points waiting list group: t0 = Baseline, t1 = pre treatment, t2 = post-treatment, t3 = 16 weeks follow-up, t4 = 32 weeks follow up.

While the two groups did not differ significantly concerning CGAS scores at t0, the CGAs score of the treatment group increased from t0 to t1 and was significantly higher than that of the waiting list group at t1. A graphical representation of the CGAS scores for every assessment time point is shown in Figure 3.

For the COIS-R child-rated and parent-rated version, as well as for the CBCL total score, there was a significant decrease in the scores from t0 to t1 independently of the group.

### Stability of treatment effects

#### Primary outcomes

##### Children’s Yale-Brown Obsessive Compulsive Scale

A mixed two-way ANOVA with group (treatment group, waiting list group) and time (post-treatment, follow-up I, follow-up II) for CY-BOCS scores showed a significant effect of time [*F* (2, 80) = 78.76, *p* = 0.020], whereas the effect of group [*F* (1, 40) = 3.20, *p* = 0.081] and group × time interaction [*F* (2, 80) = 13.78, *p* = 0.432] were not significant. The effect of time was due to a decrease in CY-BOCS scores at follow-up II compared to follow-up I [*t* (41) = 2.68, *p* = 0.011] and at follow-up II as compared to post-treatment [*t* (41) = 2.15, *p* = 0.037]. The difference between post-treatment and follow-up I was not significant [*t* (41) = 0.11, *p* = 0.915].

##### Clinical global impressions-severity

The mixed two-way ANOVA with group (treatment group, waiting list group) and time (post-treatment, follow-up I, follow-up II) for CGI-Severity scores showed no significant effect of time [*F* (2, 80) = 1.85, *p* = 0.175], group [*F* (1, 40) = 2.96, *p* = 0.093], or group x time interaction [*F* (2, 80) = 0.07, *p* = 0.888].

##### Clinical global impressions-improvement

In the treatment group, participants’ condition was rated as much better or very much better at follow-up I in 20 of 28 (71%) and at follow-up II in 20 of 28 (71%). In the waiting list group, this was the case for 17 of 19 (89%) at follow-up I, and also at follow-up II (17/19; 89%).

##### Treatment remission and response

Table 4 shows participants‘ response and remission rates in both groups for the measurement time points post-treatment to follow-up II for those who completed treatment.

**TABLE 4:** Response and remission rates for participants who completed treatment.

Percent of treated participants (included sample)						
	Response criteria fulfilled			Remission criteria fulfilled		
	Post-treatment	Follow-up I	Follow-up II	Post-treatment	Follow-up I	Follow-up II
Treatment group	79% (22/28)	71% (20/28)	68% (19/28)	64% (18/28)	67% (18/28)	68% (19/28)
Waiting list group	100% (19/19)	79% (15/19)	89% (17/19)	63% (12/19)	79% (15/19)	79% (15/19)

Only participants for whom the results of all primary outcome measures were available at all measurement time points are listed as responders and remitters.

#### Secondary outcomes

Again, p was set at 0.001 for these analyses. An overview of the analysis results for all secondary outcome measures for the time points post-treatment, follow-up I and follow-up II can be found in Table 5.

**TABLE 5:** Statistical analyses of secondary outcome measures—stability of effects.

	Mixed two-way ANOVA with the factors group (treatment, waiting list) and time (post-treatment, follow-up I, follow-up II).		
Measure	ME time	ME group	IA Time x Group
*Clinician-rated measure*			
CGAS	*F* (2, 116) = 3.92, *p* = 0.037	*F* (1, 58) = 2.13, *p* = 0.188	*F* (2, 116) = 1.77, *p* = 0.268
*Child-rated measures*			
YSR	*F* (2, 116) = 2.78, *p* = 0.229	*F* (1, 58) = 0.60, *p* = 0.601	*F* (2, 116) = 0.55, *p* = 0.649
SCARED	*F* (2, 116) = 2.74, *p* = 0.242	*F* (1, 58) = 0.43, *p* = 0.627	*F* (2, 116) = 1.03, *p* = 0.485
COIS-RC	*F* (2, 116) = 4.18, *p* = 0.053	*F* (1, 58) = 2.73, *p* = 0.252	*F* (2, 116) = 0.84, *p* = 0.529
KINDL	*F* (2, 116) = 1.26, *p* = 0.461	*F* (1, 58) = 2.60, *p* = 0.235	*F* (2, 116) = 0.77, *p* = 0.559
DIKJ	*F* (2, 116) = 4.58, *p* = 0.075	*F* (1, 58) = 1.13, *p* = 0.431	*F* (2, 116) = 0.68, *p* = 0.583
*Parent-rated measures*			
CBCL^a^	*F* (2, 116) = 8.66, *p* = 0.003	*F* (1, 58) = 1.07, *p* = 0.390	*F* (2, 116) = 0.94, *p* = 0.488
SCARED	*F* (2, 116) = 4.79, *p* = 0.051	*F* (1, 58) = 1.37, *p* = 0.371	*F* (2, 116) = 1.03, *p* = 0.448
COIS-RP	*F* (2, 116) = 5.89, *p* = 0.029	*F* (1, 58) = 2.10, *p* = 0.279	*F* (2, 116) = 1.45, *p* = 0.372
KINDL	*F* (2, 116) = 4.88, *p* = 0.075	*F* (1, 58) = 1.84, *p* = 0.304	*F* (2, 116) = 1.88, *p* = 0.246
ULQUIE	*F* (2, 116) = 0.83, *p* = 0.522	*F* (1, 58) = 13.11, *p* = 0.007	*F* (2, 116) = 0.81, *p* = 0.549

^a^*T*-values. Significant values, defined as *p* ≤ 0.001, are in bold. ME Time, Main Effect Time; ME Group, Main Effect Group. IA Time × Group = Interaction of Time × Group.

No significant effect of time, group, or time x group interaction was found in any of the outcome measures in the ANOVAs.

### Treatment satisfaction, feasibility, and implementation

For both groups together, the mean score for participants’ satisfaction with the treatment, measured with the CSQ-8, was *M* = 28.69 (*SD* = 3.78). Information on the individual items of the CSQ-8 can be found in Table 6.

**TABLE 6:** Rates of perceived benefit from treatment by patients (client satisfaction questionnaire—CSQ-8).

Item	M (SD)
(1) How would you rate the quality of care you have received?	3.61 (0.62)
(2) Did you get the kind of help you wanted?	3.55 (0.67)
(3) To what extent has the program met your needs?	3.52 (0.59)
(4) If a friend needed similar help, would you recommend the program to him/her?	3.60 (0.54)
(5) How satisfied are you the amount of help you have received?	3.67 (0.68)
(6) Has the help you have received helped you to deal more effectively with your problems?	3.64 (0.53)
(7) In overall, general sense, how satisfied are you with the help you have received?	3.64 (0.57)
(8) If you were to seek help again, would you come back to our program?	3.45 (0.81)

Anchors for Likert scale by question were as follows: Question (1) 4 = Excellent, 3 = Good, 2 = Fair, 1 = Poor; Questions (2), (4), and (8) 1 = No, definitely not, 2 = No, not really, 3 = Yes, generally, 4 = Yes, definitely; Question (3) 4 = Almost all of my needs have been met, 3 = Most of my needs have been met, 2 = Only a few of my needs have been met, 1 = None of my needs have been met; Question (5) 1 = Quite dissatisfied, 2 = Indifferent or mildly dissatisfied, 3 = Mostly satisfied, 4 = Very satisfied; Question (6) 4 = Yes, it helped a great deal, 3 = Yes, it helped somewhat, 2 = No, it didn’t really help, 1 = No, it seemed to make things worse; Question (7) 4 = Very satisfied, 3 = Mostly satisfied, 2 = Indifferent or mildly dissatisfied, 1 = Quite dissatisfied.

Based on the Final Therapy Evaluation Questionnaire conducted after the treatment, more than 90% of parents and participants reported that they liked that the therapy was conducted *via* the internet. All parents reported having a good understanding of what to do to support their children against OCD. Similarly, at the end of the treatment, all children reported having a good understanding of how to manage their OCD symptoms and how the exposure exercises work. Regarding the usability of the video conferencing program, approximately 90% stated that it worked well. The results of the final therapy evaluation can be found in detail in Table 7.

**TABLE 7:** Final therapy evaluation.

Item	Evaluation topic	Parents agreed in % (n/assessed sample)	Children agreed in % (n/assessed sample)
	**Acceptance internet-based therapy**		
1) I liked it, that the therapy was carried out via the internet.		98% (40/41)	90% (36/40)
2) I think a therapy without the internet, where I had face-to-face contact with the therapist, would have suited me better.		9% (4/43)	19% (8/41)
3) I found it useful that the worksheets were exchanged and edited via the cloud.		100% (41/41)	90% (36/40)
4) I found it useful to have the app for feedback.		66% (27/41)	77% (30/39)
	**Satisfaction**		
5) If a child from my circle of acquaintances also had a problem with OCD, I would recommend the internettherapy to him/her/the parents.		93% (38/41)	98% (39/40)
	**Therapy scope**		
6) My child/I had just the right number of therapy sessions, to learn how to conquer the compulsions.		60% (25/42)	79% (33/42)
7) My child/I would have needed more therapy sessions to learn how to get rid of OCD.		45% (19/42)	38% (15/42)
8) The amount of parent counseling was spot on.		86% (36/42)	-
9) I would have liked to have more parent counseling.		21% (9/42)	-
	**Psychoeducation**		
10) I have a good understanding of what I can do to support my child against OCD.		98% (40/41)	-
11) I have well understood how the exposure exercises work.		100% (42/42)	100% (42/42)
12) I understood well what OCD is.		98% (41/42)	100% (42/42)
	**Change**		
13) The OCD-symptoms are weaker than before the treatment.		90% (38/42)	93% (39/42)
14) Family life has improved since the treatment.		91% (39/43)	82% (31/38)
	**Therapeutic alliance**		
15) I was able to trust the therapist.		100% (43/43)	100% (42/42)
16) The therapist was interested in me/us and my/our problems.		100% (43/43)	100% (42/42)
	**Usability technical equipment**		
17) I found it difficult to use the program for video calls on the computer.		12% (5/43)	10% (4/42)
18) The videoconference program worked well.		91% (39/43)	90% (38/42)
19) We had to interrupt therapy or started later because the videoconference program didn’t work.		11% (5/43)	24% (10/42)

Items rated on a four-point Likert scale where 1 = “I agree,” 2 = “I rather agree,” 3 = “I rather disagree,” and 4 = “I don’t agree.” We have taken the answers 1 and 2 as agreement as shown in the table.

The assessment of the feasibility and implementation of the therapy from the therapists’ perspective is shown in Table 8.

**TABLE 8:** Implementation of manual content—Summary therapist feedback form (STFF).

Item	M (SD)
How easy was it to understand the content of the manual?	6.59 (0.60)
How easy was it to conduct the treatment as outlined by the manual?	5.90 (1.07)
How user-friendly were the treatment materials?	5.87 (0.66)
Did the manual allow for enough flexibility?	4.79 (0.98)
Did you feel the 14 sessions were sufficient to accomplish all of the treatment goals?	4.62 (2.06)
Where there any unnecessary elements included in the manual?	1.51 (0.68)
Where there any important elements missing from the manual?	2.87 (1.28)

Items rated on a seven-point Likert scale where 1 = “Not at all,” 4 = “Somewhat,” and 7 = “Very much”.

### Adverse events

During treatment, one participant experienced a significant increase in OCD symptoms. Due to the associated severe impairment in everyday life, inpatient treatment was initiated, ending the study intervention. A more direct relationship between the deterioration and the treatment could not be established. In all other participants who had undergone the treatment, there were no incidents that could be classified as adverse events.

## Discussion

The primary focus of our study was to examine the effectiveness of internet-based CBT for children and adolescents with OCD. The results of previous studies suggest that videoconferencing therapy for pediatric OCD is feasible (26, 27, 54). However, as we noted in more detail in the introduction, previous work has used neither a sample of this size nor such a long follow-up period. The largest sample consisted of 31 participants (27), and the longest follow-up period was 6 months (26). Although other studies used innovative ideas for using computer-based techniques (e.g., interactive computer games to enhance children’s understanding of treatment concepts) (26), both the use of technological devices (e.g., a tablet) and digital applications (therapy documents in the cloud, a mobile assessment application) in our study go far beyond the scope of previous ones. Finally, the transformation of a face-to-face therapy manual into a feasible online version is also a novel feature of the current study.

To assess the effectiveness of this approach, CY-BOCS outcomes of a group of participants who began treatment immediately after enrolment in the study were compared with those of a waiting list control group after the end of the waiting period. As we expected, OCD symptoms significantly decreased in the treatment group compared to the waiting list group over the same period. The effect size for the between-group comparison of CY-BOCS scores at time t1 (treatment group = post-treatment; waiting list group = end of waiting period) was large, with a value of *d* = 1.63. After having received treatment, participants in the waiting list group also showed a significant decline in OCD symptoms. Indeed, in both groups, after treatment, the mean CY-BOCS scores were well below the cut-off value (CY-BOCS total score ≥ 16). This decline in symptoms continued in both groups after the completion of the study as demonstrated by a decrease in OCD symptoms from post-treatment to follow-up II. Immediately after treatment, 64% of participants in the treatment group met the criteria for remission, in the waiting list group, this was 63%. This rate also remained stable during the follow-up examinations, and even increased in the waiting list group. The response criteria were met by 79% of participants in the treatment group at the post-treatment measurement time point, and by all participants in the waiting list group.

The treatment approach we adopted was found to be effective for treating mild to moderate OCD. The decrease in OCD symptoms in our study align with the results from two other randomized controlled trials which review the effectiveness of internet-based CBT in children and adolescents with OCD, where therapy sessions were conducted *via* video conferencing (26, 27). E/RPs were a central treatment element and, as far as technically possible, were accompanied therapeutically in all three studies in real time in the home environment on a computer screen. The severity of OCD symptoms at pre-treatment assessment was also comparable. Nevertheless, before further discussing the comparison of OCD symptom change, it is important to first mention the differences between interventions. In Storch et al. (27), the treatment was more compressed (14 sessions in 12 weeks) compared to our approach, and in Comer et al. (26), the involvement of parents in the therapy and their training as coaches for their children was significant due to the participants‘ young age.

In the study from Storch et al. (27), the between-group effect size (treatment vs. waiting list) was *d* = 1.36 at the post-treatment measurement time point, and the remission rate was 56% (criteria: severity rating ≤ 3 on Anxiety Disorders Interview Schedule for DSM-IV (55) and CY-BOCS total score ≤ 10). Eighty-one percent were classified as treatment responders (criteria: CGI-Improvement = 1 or 2 and ≥ 30% reduction in CY-BOCS total score). The within-group effect size (pre- vs. post-treatment) reported in Comer et al. (26) was *d* = 1.53. The rate of those who no longer met the criteria for an OCD diagnosis after the end of treatment was slightly over 63% (determined *via* Anxiety Disorders Interview Schedule for DSM-IV). Almost 73% were classified as responders (criteria: CGI-Improvement = 1 or 2). Therefore, in the current study, we actually achieved slightly greater improvements in terms of the magnitude of change in OCD symptoms, although comparabality is not entirely given for the reasons stated above. This improvement is even more valid when compared with a study using an internet-based form of CBT in which the children and adolescents largely completed treatment modules independently over a period of 12 weeks but had regular contact with a clinician by mail or telephone (56). The average time spent by the clinician per patient per week, was 17.5 min, much less than for the video-based approaches (56). The effect size in this study between the treatment group and wait-list group was *d* = 0.69 at the measurement point at post-treatment/end of the waiting period. The remission rate was 15% (criteria: CGI-Severity = 1 or 2 and CY-BOCS total score ≤ 12), as responders classified were 27% (criteria: CGI-Improvement = 1 or 2 and ≥ 35% reduction in CY-BOCS total score). As a first interim conclusion, it can be stated that our study adds substantial evidence to support the effectiveness of internet-based CBT for children and adolescents with OCD. Finally, these results align with other studies on internet-based psychotherapy in adults with OCD (57, 58).

A comparison of our results on OCD symptom decrease to those from face-to-face interventions, where CY-BOCS baseline scores were in a similar range, yields further remarkable insights. In the Pediatric OCD Treatment Study (POTS (59), the effect-size within the CBT treatment arm (pre- vs. post-treatment) was 1.35 (60). The remission rate at the same time point was nearly 40% (criterion CY-BOCS total ≤ 10); information on the number of responders was not available for us. In the Nordic Long-term OCD Treatment Study NordLOTS (60) the within-group effect size from baseline to post-treatment was *d* = 1.58. and the remission rate for the same time point was 39% (criterion CY-BOCS total score ≤ 10). Almost 73% were classified as treatment responders (criterion: CY-BOCS total score ≤ 15). Overall, it can be concluded that the effectiveness shown in our study is at least on the same level as that found in the face-to-face treatment studies.

Beyond the observation of the treatment effectiveness, the course of change is also interesting. The fact that the CY-BOCS scores once again decreased significantly after the post-treatment measurement time point is not a phenomenon found consistently in the literature and is therefore noteworthy. It is possible that the 3 months to follow-up measurement frequently chosen in studies is too short and that further reductions in OCD symptoms do not become significant until after this time. Our own results, in addition to those of other studies (26, 61), support this interpretation. Therefore, the question of the follow-up periods required for internet-based treatments to fully capture the long-term treatment effects should be further explored.

Due to the severe impairment in various areas of daily life in subjects with OCD, the level of psychosocial functioning of the patients is of particular interest. After treatment, the psychosocial functioning level of the participants improved in both groups of this study. The improvement in psychosocial functioning in addition to the decrease in OCD symptoms is a consistent finding that has been reported in other studies of technology-based CBT for pediatric OCD (26, 49, 62). The effects found in our study are in the upper range of what has been observed in these studies.

Unlike OCD symptoms, the child- and parent-rated secondary outcome measures showed few significant changes. From pre- to post-treatment/end-of-waiting period, there was a significant decrease in scores on the COIS-R and CBCL independently of treatment. We would have expected this specifically with the COIS-R. One possible explanation is the version of this measure we used. The items were translated into German by our group, but no values on validity and reliability of this German version are available. It is noteworthy that the average baseline values, rated by participants and parents, compared to our own preliminary study (28) and the study of Storch et al. (27) are below the values collected there. However, the other parameters used to determine the severity of OCD (CY-BOCS, CGI-S) are comparable to the current ones. A review of the German-language version seems reasonable.

Even though our treatment approach focused exclusively on OCD symptoms, the absence of these effects was not necessarily expected. Studies have shown that depressive symptoms (63, 64), in addition to anxiety symptoms (65) decrease under face-to-face CBT for children and adolescents with OCD. On the other hand, in a video-based CBT for OCD comparable in treatment approach and sample, the treatment group did not outperform a waiting list control in reducing anxiety and depression symptoms after having received treatment (27). Furthermore, the course of improvement in secondary anxiety and depression symptoms appears to differ from each other and, most importantly, to be independent of the reduction in OCD symptoms (66). There is also a lack of conclusive understanding of which components of CBT for OCD address anxiety and depression symptoms and to what extent. There is a need for further research to develop a more advanced understanding of the mechanisms underlying the transfer of CBT techniques to non-OCD symptoms. This is even more true for video-based treatments. Regarding our study, it can be noted that on a purely descriptive level, there is a treatment-associated decrease in mean scores for the self- and parent-rated outcome measures capturing anxiety and obsessive-compulsive symptoms (see Table 2).

Accordingly, our study delivers insights into the effectiveness of internet-based CBT for OCD. Nevertheless, the limitations of our study should also be noted. The choice of a waiting group as a control condition enables us to demonstrate that our approach led to a reduction in OCD symptoms. Furthermore, the results can be compared descriptively with those of face-to-face studies. However, a statement as to whether the internet-based treatment is actually equal to the well-established face-to-face CBT for OCD in terms of efficacy cannot be made. The next step is to conduct studies in which the treatment with face-to-face CBT is the control condition or other therapy approaches such as medication or self-help. But more effective or not, we think that internet-based CBT delivers treatment access for the patients and also the option to treat symptoms at home which has often the highest relevance for these patients.

Furthermore, it must be noted that, even though the majority of participants stated that they had sought inclusion in the study due to a lack of local therapy offers, it can nevertheless be assumed that these families were more open than average to internet-based therapy and that the sample was, thus, not fully representative of all children with OCD concerning their attitudes toward digital elements in therapy. The generalizability of the results to all children and adolescents with OCD may therefore, be limited. However, it is conceivable that the group of participants for whom digital treatment approaches represent something normal may become larger in the future. Indeed, the COVID-19 pandemic has acted as a catalyst for digitalization in healthcare (67), and this will most likely lead to digital interventions becoming an integral part of treatment and participants becoming more familiar with them.

It should also be mentioned that, despite extensive prior advice not to do so, families very occasionally made statements during follow-up assessments that revealed their group membership to the investigator.

The educational level of the parents in our sample was very high, and this was even more true for the mothers. In determining socioeconomic position, the educational level of parents is usually included as one aspect. Although studies on the association between socioeconomic position and health service utilization have reached different conclusions (68), there is evidence that families with a high socioeconomic position are more likely to visit specialized centers such as ours (69). In light of this, it makes sense to apply and evaluate our approach in routine health care as well.

The transfer of our approach into clinical practice is possible in principle. However, it should be noted that the purchase of the tablets and smartphones we distributed to the families is associated with not inconsiderable costs. Most healthcare institutions would presumably lack the corresponding financial resources. This represents a major hurdle, for the implementation of our treatment approach in routine care. To overcome this, it is necessary to design the applications technically in such a manner that they can be used on the families‘ end devices and no additional devices have to be purchased. According to our experience so far, this appears technically feasible.

It should be noted that there are also challenges during internet-based psychotherapy. Due to the limited screen area and the reduced visual channels, it is more difficult for the therapist to assess to what extent the participant is emotionally impaired or if the participant shows avoidance behavior. This could be resolved by the use of 180°C or even 360°C webcams, which offer a larger field of view. Furthermore, by using different sensors, the therapist could receive comprehensive and synchronous information regarding the participant’s current level of arousal or discomfort and react to it. Specifically, the measurement of heart rate and heart rate variability *via* ECG sensors should be considered. These can be worn by the participant *via* a chest strap and transmitted *via* Bluetooth to a mobile device that would then forward the values to the therapist. Another sensor element could be eye-tracking glasses, which could provide information about the participant’s gaze focus *via* a field camera and could help to prevent avoidance behavior during exposures. A corresponding project has already been planned in our department and is currently in the trial phase.

Technology-based treatment approaches might also be useful for other psychiatric conditions. Further studies investigating blended designs with a combination of face-to-face and internet therapy may be a beneficial next step. Furthermore, studies focusing on stepped-care designs to unravel the optimized and individualized therapy conditions for participants, including more or less intensive modules of face-to-face psychotherapy, internet-based psychotherapy, self-help elements, or medication, are warranted.

In summary, this study has demonstrated that internet-based CBT is effective for treating children with mild to moderate OCD. It enables these children to receive specialized state-of-the-art therapy regardless of their place of residence- and even enables the treatment of symptoms by therapist-guided exposures with response prevention at the location the symptoms typically occur, which is frequently at the child’s home. The implementation of exposure exercises in the living environment may increase the ecological validity of the therapy (70), which may, consequently, have a reinforcing effect on the effectiveness of the treatment. Further studies are necessary to draw conclusions regarding this reinforcing effect.

Overall, our study extends the evidence for internet-based CBT approaches to be effective for treating OCD in children and adolescents, making it a viable method for providing access to adequate treatment.

## Data availability statement

The raw data supporting the conclusions of this article will be made available by the authors, without undue reservation.

## Ethics statement

The studies involving human participants were reviewed and approved by Ethics Committee of the Medical Faculty of Tübingen, Germany. Written informed consent to participate in this study was provided by the participants’ legal guardian/next of kin.

## Author contributions

KH, CH, AH, AA, JK, AP, UW, RA, HL, TR, and AC made substantial contributions to the conception and design of the work, contributed to the analysis of the data, and contributed to the interpretation of the data. KH was mainly responsible for the first draft of the manuscript which was then revised by all other authors. All authors approved the final manuscript.

## Footnotes

1 ANOVAs revealed, neither age nor sex had influence on CY-BOCS scores at any timepoint or change of scores over time.

## Abbreviations

CBCL/16-18R: Child Behavior Checklist
CFT 20-R: Basic Intelligence Test Scale 2-Revised
CGAS: Children’s Global Assessment Scale
CGI-S: Clinical Global Impressions-Severity
CGI-I: Clinical Global Impression scale-Improvement
COIS-R: The Child Obsessive-Compulsive Impact Scale-Revised
CSQ-8: Client Satisfaction Questionaire-8
CYBOCS: The Children’s Yale-Brown Obsessive Compulsive Scale
DIKJ: Depressionsinventar für Kinder und Jugendliche
ECG: Electrocardiography
E/RP: Exposure with Response Prevention
iCBT: Internet-based cognitive behavioral therapy
KINDL: Questionnaire for Measuring Health-Related Quality of Life in Children and Adolescents
K-SADS -PL: The Schedule for Affective Disorders and Schizophrenia for School-Age Children Present and Lifetime Version
OCD: Obsessive-compulsive disorder
CBT: Cognitive behavioral therapy
RCT: Randomized controlled trial
SCARED: Screen for Child Anxiety Related Emotional Disorders
STFF: Summary Therapist Feedback Form
ULQIE: Lebensqualitäts-Inventar für Eltern chronisch kranker Kinder
YSR/11-18R: The Youth Self Report.

## Appendix

**TABLE A1:** Original data of primary and secondary outcome measures.

Unadjusted mean ± standard deviation (sample)									
	Treatment group				Wait-list group				
Measure	Baseline assessment (t0)	Post-treatment (t1)	Follow-up 1 (t2)	Follow-up 2 (t3)	Baseline assessment (t0)	End of waiting period (t1)	Post-treatment (t2)	Follow-up 1 (t3)	Follow-up 2 (t4)
*Clinician-rated measures*									
CY-BOCS	24.03 ± 2.54 (30)	9.96 ± 9.04 (28)	10.48 ± 8.46 (27)	8.35 ± 8.26 (26)	25.07 ± 2.07 (30)	23.64 ± 3.37 (22)	8.80 ± 8.38 (20)	6.85 ± 6.39 (20)	4.88 ± 6.54 (24)
CGAS	60.20 ± 10.48 (30)	83.04 ± 14.48 (28)	82.07 ± 11.53 (27)	83.62 ± 14.81 (26)	60.03 ± 10.05 (30)	64.23 ± 10.46 (22)	85.60 ± 11.78 (20)	86.55 ± 8.20 (20)	90.04 ± 8.87 (24)
CGI-S	4.93 ± 0.52 (30)	2.29 ± 1.56 (28)	2.33 ± 1.54 (27)	2.12 ± 1.48 (26)	5.07 ± 0.37 (30)	4.77 ± 0.53 (22)	2.05 ± 1.47 (20)	1.90 ± 1.07 (20)	1.50 ± 0.72 (24)
CGI-I		1.89 ± 1.17 (28)	1.81 ± 1.18 (27)	1.62 ± 1.02 (26)		3.86 ± 0.89 (22)	1.50 ± 1.15 (20)	1.35 ± 0.67 (20)	1.21 ± 0.42 (24)
*Child-rated measures*									
YSR	61.92 ± 19.91 (25)	47.27 ± 15.46 (22)	44.35 ± 15.90 (17)	42.56 ± 22.97 (16)	58.44 ± 17.98 (25)	54.10 ± 17.08 (20)	47.69 ± 17.35 (16)	47.87 ± 23.22 (15)	45.83 ± 23.86 (12)
SCARED	21.00 ± 13.44 (27)	13.56 ± 10.81 (25)	14.84 ± 9.10 (19)	12.88 ± 10.43 (16)	21.03 ± 11.52 (27)	17.48 ± 11.24 (21)	16.18 ± 10.10 (16)	14.93 ± 14.46 (15)	11.60 ± 10.54 (15)
COIS-RC	19.70 ± 17.04 (30)	7.24 ± 9.95 (25)	4.79 ± 6.89 (19)	6.44 ± 12.27 (16)	17.46 ± 10.90 (28)	11.76 ± 8.46 (21)	4.19 ± 3.83 (16)	4.07 ± 7.21 (15)	1.93 ± 4.35 (15)
DIKJ	14.36 ± 10.11 (28)	10.46 ± 8.59 (26)	7.84 ± 7.85 (19)	7.07 ± 6.65 (15)	14.85 ± 7.62 (27)	10.95 ± 8.53 (19)	8.86 ± 7.47 (14)	6.71 ± 5.68 (14)	7.42 ± 5.70 (12)
KINDL	70.40 ± 12.64 (30)	73.91 ± 11.26 (26)	72.81 ± 12.91 (18)	73.58 ± 13.68 (15)	68.69 ± 10.93 (28)	73.84 ± 11.31 (21)	77.59 ± 10.70 (16)	76.41 ± 12.39 (15)	78.94 ± 10.41 (12)
*Parent-rated measures*									
CBCL^a^	63.77 ± 7.75 (30)	56.70 ± 11.13 (27)	56.35 ± 10.47 (20)	52.53 ± 12.31 (17)	64.22 ± 6.97 (27)	60.67 ± 8.46 (21)	55.00 ± 8.49 (16)	52.29 ± 8.71 (14)	52.00 ± 7.07 (14)
SCARED	20.28 ± 13.21 (29)	15.52 ± 10.37 (25)	15.35 ± 10.89 (20)	12.76 ± 13.20 (17)	20.44 ± 8.35 (27)	17.00 ± 9.15 (20)	14.56 ± 10.27 (16)	11.60 ± 10.07 (15)	10.87 ± 7.60 (15)
COIS-RP	25.87 ± 18.10 (30)	11.32 ± 12.75 (25)	12.10 ± 18.99 (20)	8.63 ± 12.58 (16)	22.70 ± 11.98 (27)	14.86 ± 11.97 (21)	11.81 ± 18.25 (16)	6.47 ± 7.51 (15)	3.13 ± 6.07 (15)
KINDL	64.72 ± 12.68 (29)	72.41 ± 11.71 (26)	71.63 ± 12.46 (20)	74.48 ± 11.01 (17)	62.07 ± 14.13 (28)	69.41 ± 13.30 (21)	71.58 ± 8.87 (16)	78.58 ± 6.90 (15)	77.02 ± 10.43 (15)
ULQUIE	75.89 ± 16.69 (28)	75.42 ± 16.08 (24)	71.11 ± 24.04 (18)	75.53 ± 19.15 (15)	75.56 ± 15.28 (27)	82.53 ± 6.77 (19)	86.63 ± 10.48 (16)	86.69 ± 10.40 (16)	86.07 ± 15.75 (15)

^a^*T*-Values.
